# Polypyrrole as Adsorbent in Magnetic Solid Phase Extraction for Progesterone Determination from Human Plasma

**DOI:** 10.1021/acsomega.4c05456

**Published:** 2024-09-11

**Authors:** Iara Amorim Carvalho, Camilla Fonseca Silva, Raíra da Cunha, Keyller Bastos Borges

**Affiliations:** Departamento de Ciências Naturais, Universidade Federal de São João del-Rei, Campus Dom Bosco, Praça Dom Helvécio 74, Fábricas, 36301-160 São João del-Rei, Minas Gerais, Brazil

## Abstract

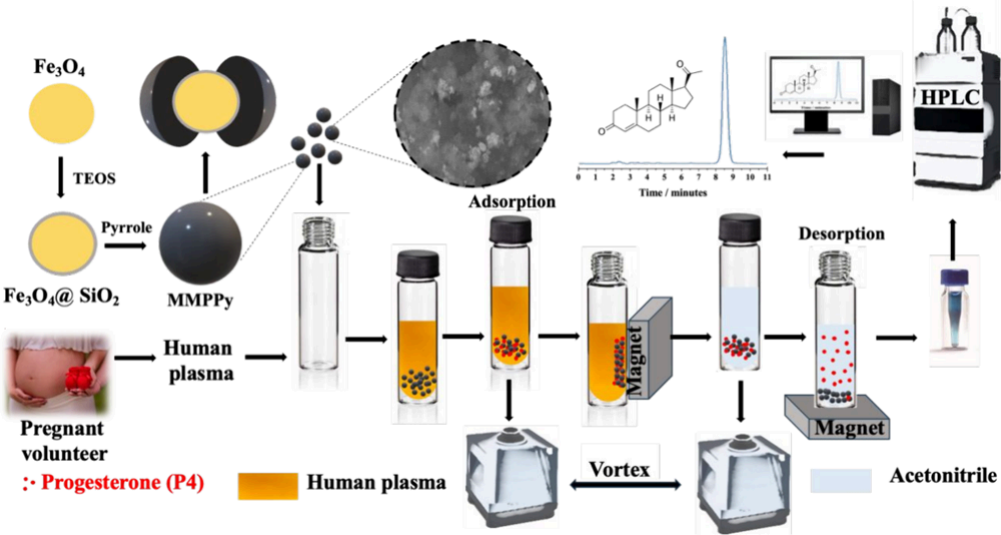

A straightforward and effective chromatographic method has been created for the analysis of progesterone from human plasma using a composite based on polypyrrole/magnetic nanoparticles in the sample preparation procedure. The quantification of progesterone is necessary due to its importance in human development and fertility. The employed conditions used acetonitrile:ultrapure water (70:30, v/v) as the mobile phase at 1.0 mL min^–1^ and an octadecyl silane column (Phenomenex Gemini, 250 mm × 4.6 mm, 5 μm) at a wavelength of 235 nm. The composite and its precursors were synthesized and properly characterized by X-ray diffraction, Fourier transform infrared spectroscopy, scanning electron microscopy/energy dispersive spectroscopy, thermogravimetric analysis, and point of zero charge. The main factors affecting the extraction recovery of progesterone from pool human plasma samples employing magnetic solid phase extraction have been studied, such as sample pH (without adjustment), sample volume (1000 μL), washing solvent (ultrapure water), eluent (acetonitrile), eluent volume (1000 μL), and amount of adsorbent (10 mg). The extraction recoveries ranged from 98% to 102%, and linearity ranged from 5 to 3000 ng mL^–1^. The correlation coefficient (*r*) was ≥0.99, and acceptable relative standard deviation (precision), relative error (accuracy), and *p*-values (robustness) were observed. Lastly, the plasma samples from pregnant women were successfully analyzed by the validated method.

## 1 Introduction

Essential functions of endogenous sex hormones include the development of female and male sexual characteristics. These hormones are produced by complex metabolic processes and are sourced from cholesterol.^1^ Female sex steroid hormones mainly include estrogens and progesterone (P4),^2^ being synthesized by the ovaries, adrenal glands, and placenta, which perform a substantial role in the maintenance of pregnancy as well as at the beginning of labor.^3^ P4 keeps the fertilized egg inside the uterus and keeps it from being ejected by the body via controlling a woman’s menstrual cycle. Early in pregnancy, it might be helpful if progesterone levels are low. P4 levels in human blood vary from 0.15 to 25 ng mL^–1^ and can increase to 230 ng mL^–1^ during pregnancy. For a pregnancy to be successfully maintained, an increase is necessary. P4 levels in human blood are likewise highest in the middle of the menstrual cycle (5–20 ng mL^–1^), lowest at the start of the cycle (∼1 ng mL^–1^), and lowest after menopause.^1−3^ In this way, measuring P4 is important for assessing reproductive health, monitoring ovulation, diagnosing fertility problems, and ensuring a healthy pregnancy.^4^

The levels of steroid hormones are typically determined through immunoassays or radioimmunoassays. These tests are still utilized in hospitals because of their low cost, minimal investment, and simplicity of use. Cross-reaction, however, is possible since these tests rely on the interaction between an antibody and an antigen, particularly in complex matrices like serum, plasma, and urine.^5^ However, since commercially available high-performance liquid chromatography (HPLC) instruments have become prevalent, the scenario has changed as they offer superior selectivity and sensitivity when compared to immunoassays,^6^ such as the method presented by Tai et al. to quantify P4 using liquid chromatography–tandem mass spectrometry (LC-MS/MS).^7^ Laszlo et al. presented a procedure for the simultaneous determination of 11 synthetic progestins in human plasma by use of high-resolution liquid chromatography–mass spectrometry (HRLC-MS).^8^

Several studies in the literature focus on determining P4 from various biological samples. However, biological matrices, which are complex and contain various interferents, require more sensitive and selective analytical methods to determine their analytes. This enhances the reliability of the analyses. Sample preparation is crucial in this scenario, as it involves isolating and concentrating specific analytes. Apart from facilitating cleaning, it should also lead to an increase in the enrichment factor of these compounds. A comprehensive analytical method encompasses different stages ranging from sample collection to data manipulation, including sample preparation and detection of the analyte using suitable instruments, such as HPLC, CE, and mass spectrometry. To ensure the effectiveness of this process, sample preparation is essential for reducing interferents without compromising the identification of analytes.^9−11^

In this context, magnetic solid phase extraction (MSPE) emerges as a sample preparation technique based on magnetic interaction. In MSPE, a magnetic adsorbent material is dispersed in a suspension or solution containing the analyte, which is adsorbed by the magnetic material after a short period of time. The magnetic material can then be separated from the solution or suspension using a magnet, followed by a washing and desorption process; then, the resulting desorbed solution can be analyzed.^12^ The introduction of magnetism in sample preparation represents an increasingly interesting and developing idea in the current research. Magnetic adsorbents, a new category of adsorbents, combine conventional adsorbents with magnetic nanoparticles, such as metal oxides (e.g., Fe_3_O_4_).^13^ This innovative approach offers the advantage of applying an external magnetic field, allowing the rapid and easy separation of the adsorbent from water or biological matrices due to the presence of magnetic nanoparticles. Thus, the primary objective of incorporating a magnetic coating is to enable efficient isolation of the material within the matrix. This separation process is achieved through the application of a magnet, which selectively separates materials with magnetic properties from nonmagnetic components. This capability facilitates the execution of the adsorption procedure in an aqueous environment, ensuring residue-free extraction of the adsorbent material.^13,14^

Several adsorbents have been used in different sample preparation techniques. Polypyrrole (PPy) is a conductive polymer that has gained prominence due to its characteristics such as high electrical conductivity, a π-electron conjugated system along the polymer chain, large surface area, good thermal stability, and easy synthesis, making it very attractive for use as an adsorbent.^15−17^ Our research group has developed some studies with conductive polymers as adsorbents in pipette-tip solid phase extraction (PT-SPE),^18,19^ microextraction in packed sorbent (MEPS),^20^ dispersive solid phase extraction (DSPE),^21−24^ as well as MSPE.^25−27^ However, so far, no research is dedicated to analyzing P4 in the plasma from pregnant women using HPLC-UV and PPy-based mesoporous and magnetic adsorbents (MMPPy) in MSPE. This absence of studies using new adsorbents, including conducting polymers and their composites, highlights the importance and novelty of the current study, aiming to address this significant gap in the scientific literature. MMPPy has been characterized by Fourier transform infrared spectroscopy (FTIR), scanning electron microscopy (SEM), energy dispersive spectroscopy (EDS) analysis, pH of point zero charge (pH_PZC_), thermogravimetric analysis (TGA), and X-ray diffractometry (XRD). Several parameters that affected sample preparation were evaluated, and finally, after sample preparation and validation, the method was applied in the analyses of plasma from pregnant volunteers.

## 2 Results and Discussion

### 2.1 TGA

**Figure 1:**
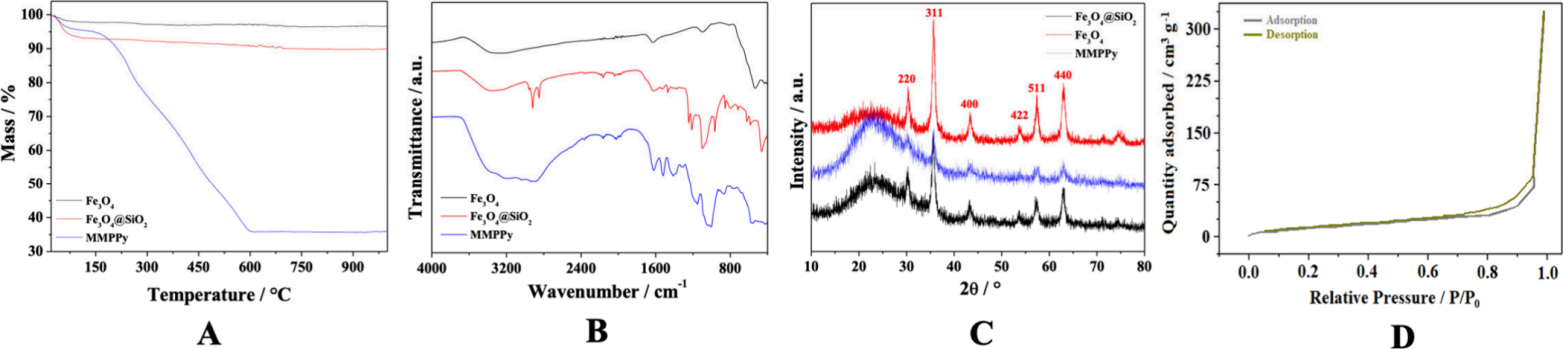
(A) TGA, (B) FTIR, and (C) XRD data of Fe_3_O_4,_ Fe_3_O_4_@SiO_2_, and MMPPy. (D) BET nitrogen adsorption–desorption isotherm plot of MMPPy.

The thermograms of Fe_3_O_4_ and Fe_3_O_4_@SiO_2_ in Figure 1A show a small loss of mass of 2% and 8%, respectively, which can be explained by the high thermal stability of Fe_3_O_4_, which has a high melting temperature. MMPPy presents two mass losses, in which the first thermal event (6% mass loss) can be explained by the decomposition of volatile compounds or the evaporation of water. The other thermal event at approximately 160 °C may be related to mass loss due to thermal decomposition of the polymer chains. Even after decomposition of MMPPy, 35% of the mass is retained even after MMPPy decomposes, which is explained by the existence of magnetic particles that do not decompose in the temperature range.^18,19,25,26^

### 2.2 FTIR

The characteristic groups of each material may be confirmed in the FTIR spectra (Figure 1B) and Table S1, i.e., Fe_3_O_4_, Fe_3_O_4_@SiO_2_, and MMPPy, which present a series of peaks consistent with the findings in the literature.^19,25^ The characteristic band of the Fe–O bond occurs at 586 cm^–1^, which was observed in the Fe_3_O_4_@SiO_2_ spectrum, as well as bands characteristic of SiO_2_, such as the bands at 1100 and 804 cm^–1^ that can be attributed to the asymmetric stretching of the Si–O–Si bond and two absorption bands: a typical band at 960 cm^–1^ linked to Si–OH stretching and one at 948 cm^–1^ associated with the Si–O stretching vibration. The bands at 1506 and 1499 cm^–1^ correspond to pyrrole’s C=C stretching. The asymmetric and symmetric C–C stretching vibrations of the pyrrole ring are represented by the absorption bands at 1553 and 1465 cm^–1^, respectively. The pyrrole ring’s C–N bond is stretching, as shown by the band at 1309 cm^–1^. C–C stretching vibrations are detected at 1180 cm^–1^. The pyrrole ring’s C–H bond vibration causes the absorption band at 1045 cm^–1^, while the C–C deformation vibration outside the ring’s plane causes the absorption band at 916 cm^–1^. It is possible to observe a predominance of PPy bands, indicating that the material was efficiently synthesized.^18,19,25,26^

### 2.3 XRD

XRD was used to assess the synthesized materials’ phases’ purity and crystallinity. Six peaks that correspond to the magnetite phase are seen in Figure 1C and are identified as the (220), (311), (400), (422), (511), and (440) crystal planes. These peaks are consistent with the findings in the literature for Fe_3_O_4_.^25^ The intensities of the peaks analyzed for Fe_3_O_4_@SiO_2_ are lower than those observed for the Fe_3_O_4_ peaks, which was expected since Fe_3_O_4_ was modified with SiO_2_. Fe_3_O_4_@SiO_2_ also exhibits a small amount of amorphous behavior, as seen by the large band that appears at the diffractogram’s commencement in the 2θ range at the 20° region. A wide band at the start of the MMPPy diffractogram is indicative of amorphous PPy. The presence of this broad peak in MMPPy suggests that Fe_3_O_4_ was encapsulated by the polymer. The final material preserves the crystalline structure of Fe_3_O_4_ and guarantees the magnetic properties of the synthesized materials.^25^

### 2.4 Textural Properties

The BET adsorption–desorption isotherm of MMPPy is depicted in Figure 1D. The adsorption isotherm curve is classified as type III by IUPAC. According to the BET study, MMPPy has a surface area of 48.1 m^2^ g^–1^ and a volume of 0.61 cm^3^ g^–1^. In addition, the inclusion of micelles (2 g of sodium dodecyl sulfate, surfactant) during MMPPy synthesis resulted in a mesoporous adsorbent with a pore size of 28.8 Å, potentially facilitating medicinal drug adsorption on the adsorbent surface. These results are in accordance with previous work of our research group.^28^

### 2.5 SEM/EDS

**Figure 2:**
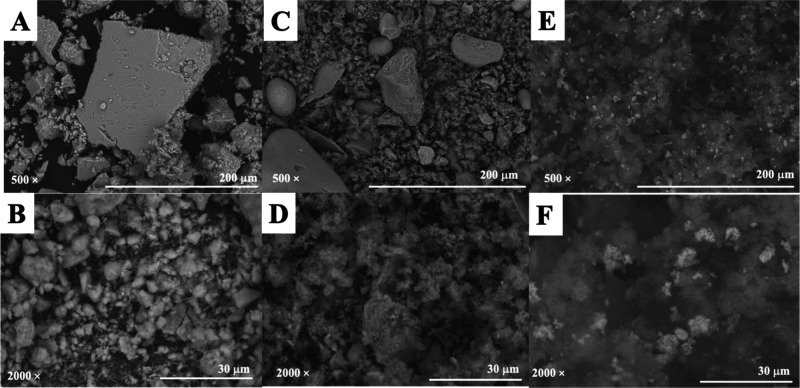
SEM images at magnifications of 500× and 2000× for (A, B) Fe_3_O_4_, (C, D) Fe_3_O_4_@SiO_2_, and (E, F) MMPPy.

Figure 2 shows the SEM images of the Fe_3_O_4_, Fe_3_O_4_@SiO_2_, and MMPPy at 500× and 2000× magnification. These materials have undefined shapes with different and irregular sizes (Figure 2A–F). The PPy synthesis modified the surface of Fe_3_O_4_@SiO_2_. In addition, these materials were characterized by EDS to evaluate the components present (Table S2). EDS analysis showed the presence of several expected elements in each material. The Fe_3_O_4_ material contains large amounts of Fe and O, as expected. Fe_3_O_4_@SiO_2_, presented, in addition to Fe and O, a large amount of Si, which can be explained by the TEOS coating. MMPPy also contains Fe, O, and Si, but in smaller quantities. A large amount of C can also be seen in the MMPPy material, which is related to the presence of C in the PPy chain.^19,25^ The presence of minor contaminants is common in this analysis, which may have come from carbon tape (contaminated or other samples), reagents used in the synthesis, and dirt on the equipment, among others.

### 2.6 pH_PZC._

Figure S1 shows that MMPPy showed a pH_PZC_ equal to 3.23. The balance of positive and negative charges on the material’s surface is zero at this pH. Because it can assess the behavior of the electrical charges on the surface of the adsorbent material, it is crucial to ascertain the pH_PZC_ of adsorbent materials prior to examining sample preparation. For instance, the surface of the adsorbent material is protonated, or positively charged, if the pH of the solution is lower than pH_PZC_, which potentially encourages the adsorption of anionic species. The surface is negatively charged and facilitates the adsorption of cationic species if the pH value is higher than that of pH_PZC_. In this case, as there is no need for pH adjustment, the adsorbent has negative charges and the analyte is in neutral form, which can generate interactions of the van der Waals type, dipole–dipole interactions, hydrogen bonds, or π–π interactions.

### 2.7 MSPE Optimization

The quantity of MMPPy, sample volume, sample pH, elution solvent, and elution solvent volume are some of the critical aspects that must be studied in order to assess the usability of MSPE coupled to HPLC-UV for the determination of P4 in human plasma. Every experiment was performed in triplicate. Prior to beginning the sample preparation, certain conditions were pre-established, including 500 μL of spiked pool human plasma at 10 μg mL^–1^, a sample pH that was left unadjusted, 10 mg of MMPPy, 500 μL of ultrapure water for washing solvent, and 500 μL of methanol as eluent. As the magnetic material is separated from the solution using a magnet, this makes the sample preparation process simpler, as it does not require filtration or centrifugation. In summary, MSPE is a sample preparation technique that has received attention due to its numerous advantages, including being environmentally friendly, easy to introduce new adsorbents, low cost, and easily automated as well as presenting a fast separation process, miniaturized technique, and efficient adsorption.^29,30^

#### 2.7.1 Washing Solvent

**Figure 3:**
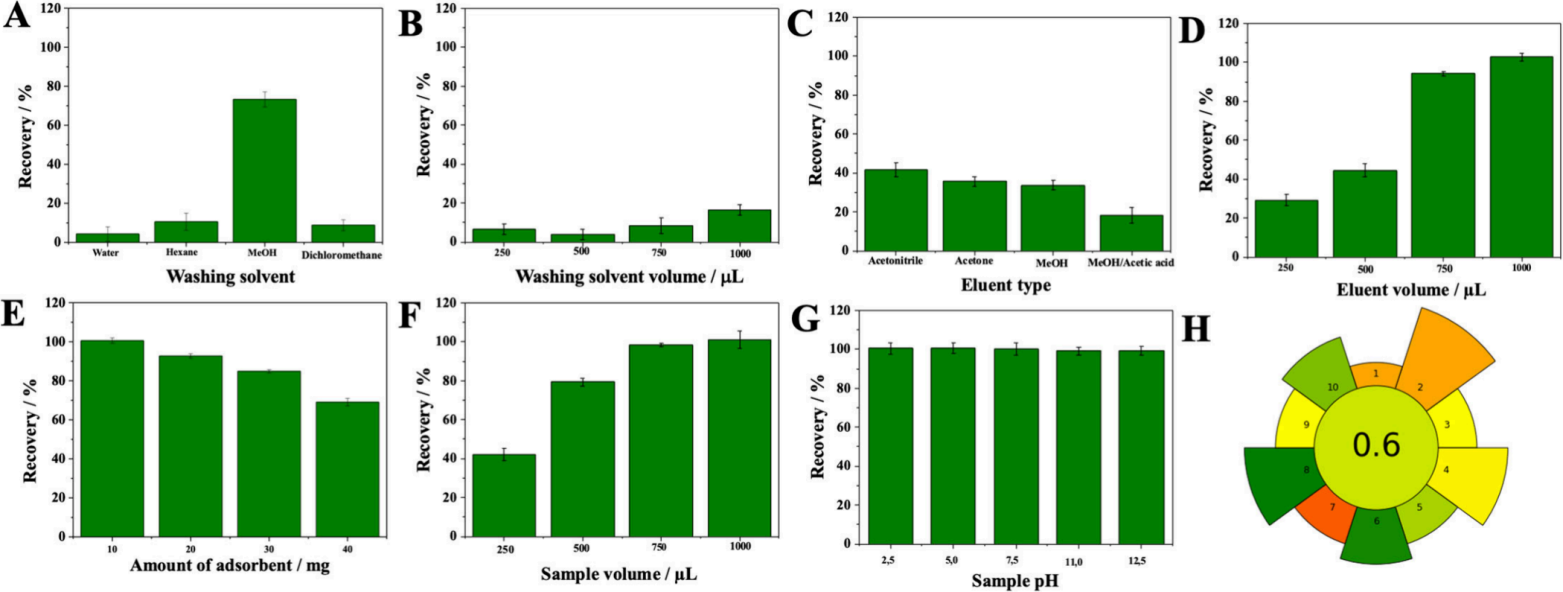
Sample preparation optimization using MMPPy in MSPE: (A) washing solvent, (B) washing solvent volume, (C) eluent type, (D) eluent volume, (E) amount of adsorbent, (F) sample volume, and (G) sample pH. (H) Graphical results from the AGREEprep analysis calculated by emulator version online.

The following washing solvents were evaluated: ultrapure water, hexane, methanol, and dichloromethane. In this step, the optimal washing solvent should effectively minimize or eradicate the presence of interferences within the sample while ensuring that it does not substantially impede the recovery of the target analytes. The solvent that eluted the least amount of analyte and eliminated the most interferents was ultrapure water, which was chosen as the washing solvent (Figure 3A).

#### 2.7.2 Washing Solvent Volume

Several washing solvent volumes were assessed to remove interferences and guarantee that the washing procedure does not elute a significant amount of the analytes. The washing solvent volumes evaluated were 250, 500, 750, and 1000 μL. The volume of 500 μL of washing solvent eluted the least amount of analyte and eliminated the most interferents (Figure 3B).

#### 2.7.3 Eluent Type

In order to effectively elute the analytes that were retained in the adsorbent material, the type of elution solvent was assessed. The following eluents were evaluated: methanol, acetone, acetonitrile, and methanol:acetic acid solution (9:1, v/v). The results are illustrated in Figure 3C. The best efficiency was achieved using acetonitrile; therefore, this was chosen as the elution solvent. This solvent probably has a similar polarity to the analyte, which justifies the high efficiency.

#### 2.7.4 Eluent Volume

The effect of the eluent volume on the extraction efficiency showed an excellent result with an increase in the volume of acetonitrile. The elution volumes evaluated were 250, 500, 750, and 1000 μL. Figure 3D shows that the recovery reached 102% when 1000 μL of acetonitrile was used.

#### 2.7.5 Amount of Adsorbent

In this study, 10, 20, 30, and 40 mg of material were evaluated. It was possible to observe that when increasing the amount of material from 10 to 20 mg, there was a slight decrease in recovery (Figure 3E). Theoretically, a larger amount of material can have more sites available to absorb more analytes until it becomes constant, but a higher amount of adsorbent demands more eluent volume. Therefore, 10 mg was chosen as the ideal amount of material because it presented a recovery of around 100% using only 1000 μL of acetonitrile.

#### 2.7.6 Sample Volume

The sample volumes evaluated were 250, 500, 750, and 1000 μL. It can be seen in Figure 3F that increasing the sample volume favors recovery. Since the 1000 μL volume achieved around 100% recovery, higher volumes were not studied.

#### 2.7.7 Sample pH

Sample pH did not present a significant effect on the P4 recoveries (Figure 3G). This behavior can be explained because P4 is a neutral drug with no ionizable atoms, i.e., it will always be in its molecular form (uncharged), interacting with the material through van der Waals, dipole–dipole, π–π, and other interactions.^31^ Thus, it was not necessary to adjust the pH of the samples. All evaluated parameters as well as optimized conditions of the sample preparation procedure are summarized in Table S3.

#### 2.7.8 Greenness Score of MSPE Procedure

The outcomes of this MSPE procedure using the AGREEprep program are displayed in Figure 3H, and the data input for each subcategory is shown in Figure S2. The final score is presented in the middle, while the 1–10 subcriteria are scattered around the inner circle, with the length of each sector signifying the weighting of the evaluated criterion. The absence of integration and automation of MSPE received the lowest marks (score = 0.19). MSPE requires some steps on sample preparation procedures such as adsorbent synthesis, adsorbent weighing, sampling, agitation, sample removal (using a magnet), washing, eluting, evaporating, reconstitution, injection, elution, eluent drying, resuspension, and injection. A score of 0.6 is regarded as being fairly green.^32,33^ The sample preparation process involves all of these steps, which can be minimized if several samples are conducted in parallel.

### 2.8 Reuse

**Figure 4:**
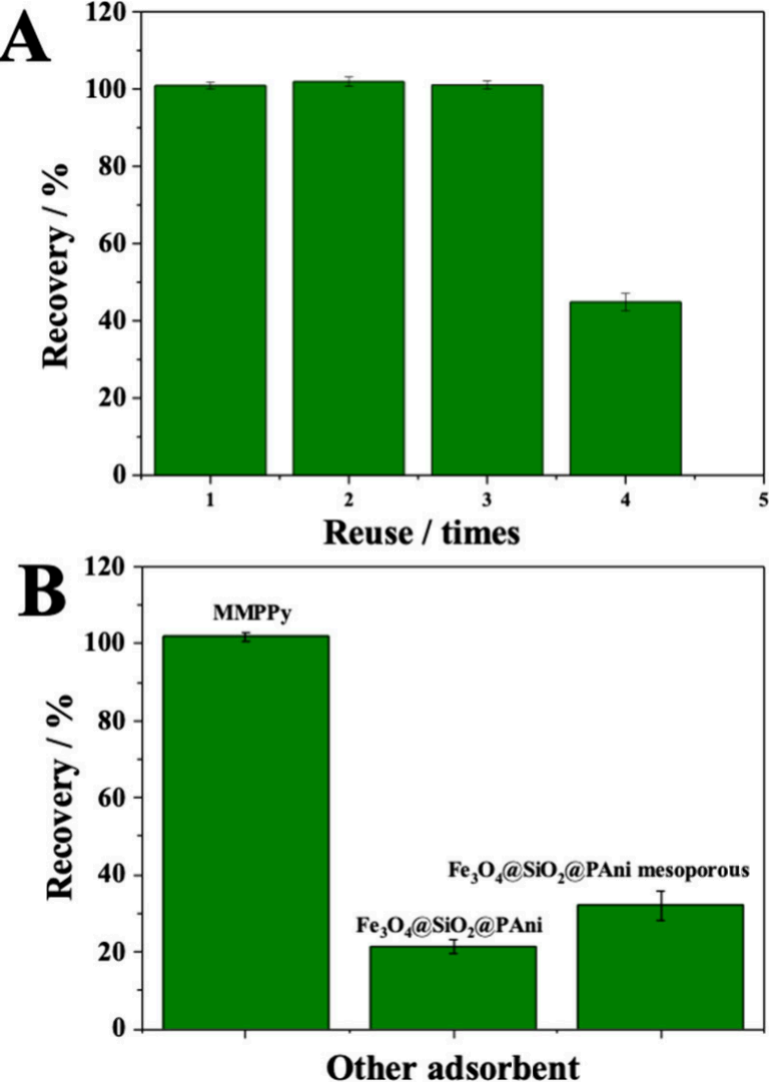
(A) Reuse (1–4×) and (B) comparison of different materials: MMPPy, Fe_3_O_4_@SiO_2_PAni, and Fe_3_O_4_@PAni mesoporous.

Following optimization, the material’s capacity to sustain P4 extraction recovery during many extractions was assessed. After extraction/washing steps (1000 μL of ultrapure water followed by 1000 μL of acetonitrile), it can be observed in Figure 4A that P4 recovery did not decrease in three reuses, and only in the fourth cycle was there a reduction of 60% in efficiency of extraction. The interaction sites of adsorbent might have been filled by matrix interfering, such as macromolecules, i.e., proteins, lipids, carbohydrates, and other components. Therefore, this adsorbent can be used up to three times.

### 2.9 Comparison with Other Materials

The same optimized sample preparation conditions were used for comparison with those of other magnetic adsorbent materials. MMPPy was compared with polyaniline-based magnetic and mesoporous adsorbent materials, i.e., Fe_3_O_4_@SiO_2_/polyaniline and Fe_3_O_4_@SiO_2_/polyaniline mesoporous, both synthesized in our research group.^14^ The results are shown in Figure 4B. The adsorbent with the best recovery capacity was MMPPy, proving the effectiveness of the material proposed in this study for use in sample preparation for the analysis of P4 from human plasma.

### 2.10 HPLC-UV Method

**Figure 5:**
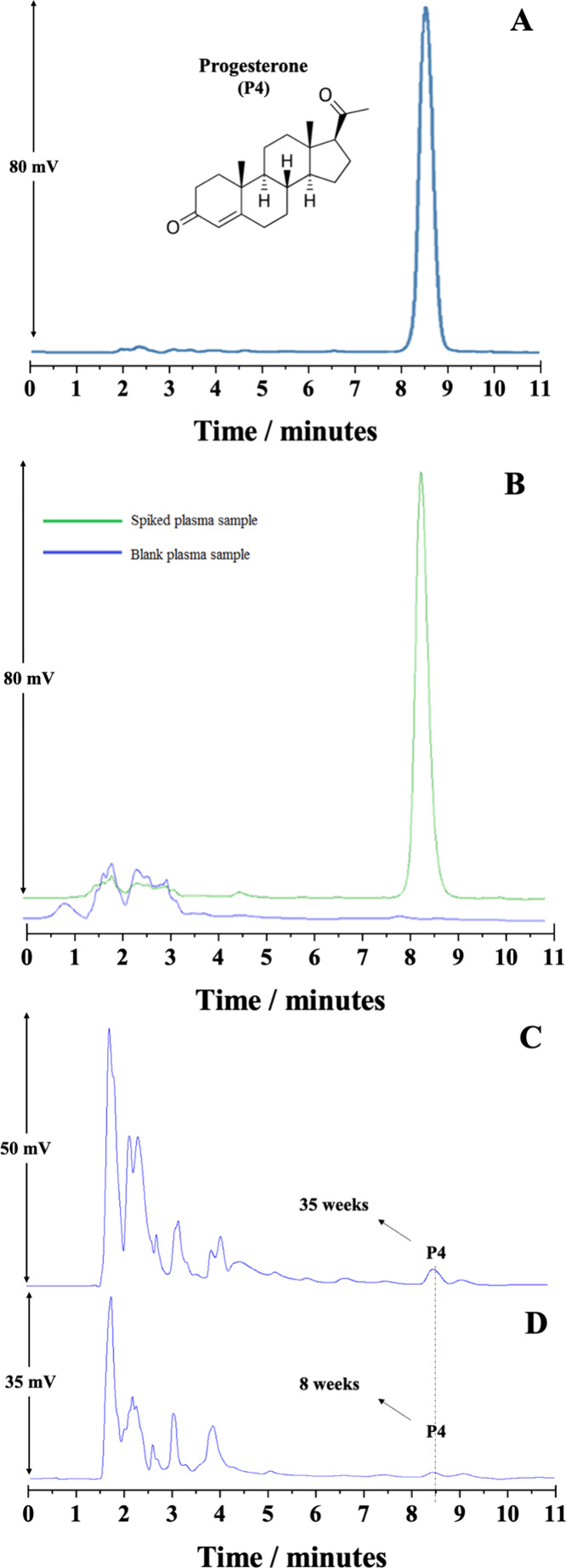
Chromatograms referring to the determination of (A) progesterone (P4) standard at 10 μg mL^–1^, (B) spiked human plasma sample at 10 μg mL^–1^ with P4 (green line) and blank pool human plasma sample (blue line), (C) 35 weeks pregnant woman plasma, and (D) 8 weeks pregnant woman plasma. Chromatographic conditions: Phenomenex Gemini C18 analytical column (250 mm × 4.6 mm, 5 μm) without temperature control, mobile phase composed of acetonitrile:ultrapure water (70:30, v/v), flow rate at 1.0 mL min^–1^, 20 μL injection volume, and detection at λ = 235 nm.

To ascertain P4 from human plasma, an easy-to-use, quick, and sensitive HPLC-UV method has been developed. The acceptable conditions were a C18 column (Phenomenex Gemini, 250 mm × 4.6 mm, 5 μm), flow rate of 1.0 mL min^–1^, and wavelength of 235 nm. Several factors, including the chromatographic column and mobile phase, were assessed. A mixture of acetonitrile and ultrapure water (70:30, v/v) was used as the mobile phase. The P4 standard solution chromatogram at 10 μL mL^–1^, obtained under the previously mentioned conditions, is shown in Figure 5A. Table S4 lists the chromatographic system’s adequacy parameters, with theoretical plates (*N*) > 2000, acceptable retention times (*t*_r_) (∼8.5 min), and asymmetry factors (*A*_F_) close to 1.0.

### 2.11 Analytical Method Validation

The validation of the devised analytical method was conducted in accordance with the guidelines provided by the literature^34^ for the analysis of biological samples, including human plasma. After obtaining acceptable chromatographic conditions for P4 determination, hydrolyzed human plasma and human plasma spiked with P4 (MSPE procedure) were analyzed to observe the presence of possible interferents from the matrix. Since human plasma is a biological matrix, it has different interferents. The selectivity study was carried out to discover if these interferents could be distinguished from the P4 chromatographic peak. The results of the analysis show that the interferents do not affect the analysis, as can be seen in Figure 5B.

The linearity data obtained by validating the analytical method are listed in Table 1. The data describe the linear equation for P4 and the respective correlation coefficients (*r*), the concentration range (ng mL^–1^), the percent relative standard deviation (%RSD) values for the slope of each of the analytical curve, the LOQ (ng mL^–1^), and %RSD of LOQ. The method was considered linear in the range of 5 to 3000 ng mL^–1^. The results obtained for the linearity are in accordance with the literature requirements, i.e., *r* > 0.98 and %RSD < 15%. In addition, the linearity was also confirmed by the *F*-test (ANOVA lack of fit), with a calculated *F*-value of 0.2 for P4 below the value of *F* tabulated (2.64). The experimentally determined LOQ was 5.0 ng mL^–1^ and showed %RSD and percent relative error (%RE) values below 20%, exhibiting acceptable accuracy and precision. The LOD was identified as 3.0 ng mL^–1^. The values referring to the intra- and interday precision are shown in Table S5. For the concentrations analyzed, it is possible to observe that the %RSD values obtained are between 1.55 and 7.76 (intraday assay)/0.16 and 3.38 (interday assay) and %RE varies between −4.39 and −14.1 (intraday assay)/–3.69 and 7.03 (interday assay). These results of %RSD and %RE are in accordance with the literature and guidelines.^34^

**Table 1:** Linearity, ANOVA Lack of Fit, and Limit of Quantification of the Analytical Method for P4

Linearity	
linear equation^a^	*y* = 6456.81*x* + 36525.15
correlation coefficient, *r*	0.9912
interval (ng mL^–1^)	5–3000
RSD (%)^b^	9.33
*F*-value^c^	0.2
LOQ	
nominal (ng mL^–1^)	5.00
analyzed (ng mL^–1^)	5.42
RSD (%)^d^	4.11
RE (%)^e^	8.45

a Calibration curves determined in triplicate (*n* = 3); *y* = *ax* + *b*, where *y* = peak area of the analyte, *a* = slope, *b* = intercept, and *x* = concentration of the measured solution (ng mL^–1^).

b %RSD = relative standard deviation percentage of the slope of the calibration curve.

c *F*_crit_ ≤ *F*_tab_ = 2.64.

d %RSD = relative standard deviation percentage of LOQ.

e %RE = relative error with an average of six repetitions.

In the robustness test, the variables were treated using one-way ANOVA statistical tests. A significance level of *p* ≥ 0.05 was adopted. Table S6 shows the chromatographic conditions and investigation intervals, as well as the %RE, %RSD, and *p*-values. The data in Table S5 show that %RSD and %RE are less than 15% and *p* > 0.05, indicating that the method is robust within the ranges studied. Therefore, the results showed that the samples were not stable after 96 h of freezing at 2500 ng mL^–1^ since the *p*-value was less than 0.05 (Table S7). These data show the importance of samples being properly analyzed within the intervals in which there were no losses in P4 concentrations.

### 2.12 Method Application

According to the optimized chromatographic conditions and the developed and validated analytical method, plasma samples from four pregnant women volunteers were analyzed. After sample analysis, plasmatic concentrations of 11.5, 38.3, 114.1, and 150.1 ng mL^–1^ were obtained for pregnancy weeks of 8, 20, 35, and 37, respectively, which is in accordance with the literature data.^35,36^ Panels C and D of Figure 5 show chromatograms referring to P4 determination from human plasma of pregnant women volunteers at 35 and 8 weeks of pregnancy, respectively.

### 2.13 Comparison with Other Methods

Table 2 presents information on some studies involving P4 determination in biological samples, such as the instrumental technique, range, recovery, sample type, LOQ, and LOD.^36−45^ It is important to note that few studies used HPLC-UV as an instrumental technique, which has inferior sensibility than other detectors such as mass spectrometry or fluorescence. It is significant to note that no research using the adsorbent material employed in this work in conjunction with the MSPE approach has been found. The sample preparation procedure archived a recovery of around 100% for P4. Most methods employed traditional sample preparation techniques, and we used a miniaturized technique. The LOD and LOQ were obtained experimentally by decreasing the concentration of analytes in human plasma samples, and the others were obtained theoretically by data from calibration curves. In addition, this sample preparation procedure presented a significant enrichment factor (*E*_F_ = 20). This demonstrates the originality of the study and its potential use in the trace-level detection of P4 in other biological materials.

**Table 2:** Literature Review on Analytical Methods for Determination of P4

analysis technique	extraction technique	sample	range (ng mL^–1^)	LOD/LOQ (ng mL^–1^)	recovery (%)	reference
LC-MS/MS	protein precipitation	human serum	-	0.03	66	(37)
				0.40		
LC-MS/MS	on line SPE	human serum	0.08–25	0.03	81.5	(38)
				0.08		
LC-MS/MS	LLE	human and mouse serum	0.05–64	0.05	100–108	(39)
				0.10		
LC-MS/MS	on line SPE	human plasma	0.08–795 (nmol L^–1^)	64 (pmol L^–1^)	89–92	(40)
				84 (pmol L^–1^)		
LC-MS/MS	LLE	human plasma	-	LOQ = 0.03	82–115	(41)
HPLC-UV	SPE	human plasma	0.1–200	LOD = 0.07	97.5	(42)
HPLC-DAD	SPE	human urine	9.5–950	0.47	81.7	(43)
				1.15		
LC-MS/MS	LLE	human saliva	0.1–50	0.05	90	(44)
				0.10		
HPLC-FLD-UV	in tube SPME	human urine	0.16–32 (ng L^–1^)	LOD = 40 (ng L^–1^)	120	(45)
LC-MS/MS	LLE	human plasma	0.2–1000	LOQ = 0.2	-	(46)
HPLC-UV	MSPE	human plasma	5–3000	1.0	100	this work

## 3 Conclusions

A polypyrrole-based mesoporous and magnetic adsorbent material named MMPPy was easily synthesized and properly characterized by XRD, TGA, FTIR, SEM, EDS, and pH_PZC_. It was employed together with a simple and efficient HPLC-UV analytical method for the determination of P4 from human plasma samples of pregnant women. The validation of the method demonstrated strong linearity, robustness, stability, and adequate precision and accuracy. MSPE was shown to be an easy-to-use, fast-moving, and adaptable method that achieves good extraction efficiency (∼100% recovery) with little solvent and adsorbent usage. Lastly, the technique produced satisfactory results when used to determine P4 from human plasma samples taken from pregnant volunteers.

## 4 Experimental Section

### 4.1 Standards, Reagents, and Solvents

A stock solution at a concentration of 1 mg mL^–1^ P4 (≥98%) (Sigma-Aldrich, St. Louis, MO, USA) was prepared, and dilutions at concentrations of 5–3000 ng mL^–1^ using HPLC grade methanol purchased from Dinâmica (Diadema, SP, Brazil) were obtained. These solutions were stored at −20 °C in the absence of light. Iron chloride hexahydrate was purchased from Dinâmica (Diadema, SP, Brazil). Iron(II) sulfate heptahydrate, anhydrous dibasic sodium phosphate (Na_2_HPO_4_, 98%), sodium hydroxide (NaOH), and anhydrous monobasic sodium phosphate (NaH_2_PO_4_, 98%) were obtained from Neon (São Paulo, SP, Brazil). Tetraethoxysilane (TEOS) was purchased from Merck (Darmstadt, Hessen, Germany). Ammonium hydroxide (NH_4_OH, 28%) was purchased from Quemis (Indaiatuba, SP, Brazil), and pyrrole (98%) was purchased from Sigma-Aldrich (St. Louis, MO, USA), which was previously purified by distillation and stored under refrigeration. Sodium dodecyl sulfate (SDS) was obtained from Isofar (Duque de Caxias, RJ, Brazil). HPLC grade methanol and acetonitrile were purchased from J. T. Baker (Mexico City, Mexico), and chloroform and dichloromethane were obtained from Dinâmica (Diadema, SP, Brazil). The water was distilled and purified by a Millipore Milli-Q Plus system (Bedford, MA, USA).

### 4.2 Instrumentation

A chromatograph from Agilent Technologies, model 1260 Infinity (Palo Alto, CA, USA), equipped with an ultraviolet (UV) detector, an isocratic pump, and a manual injector, was used to conduct the P4 analyses. Agilent Open LAB Chromatography Data System software was used to collect and handle the data. A C18 column (Phenomenex Gemini, 250 mm × 4.6 mm, 5 μm) without temperature control was used for the chromatographic separation, and the mobile phase consisted of acetonitrile and ultrapure water (70:30, v/v). An aliquot of 20 μL was injected in the HPLC equipment; 1.0 mL min^–1^ was the flow rate, and 235 nm was the detection wavelength.

The materials were put on carbon tape without any pretreatment, and SEM pictures and EDS data were obtained using a microscope (TM3000 Hitachi Analytical Tabletop, Tarrytown, NY, USA) with a voltage of 20 kV. Quantax 70 software was utilized for elemental analysis. In a thermocouple 2950 thermal analysis instrument (TA Instruments, New Castle, DE, USA), thermogravimetric analysis (TGA) was conducted under nitrogen flow conditions (50 mL min^–1^) at temperatures between 30 and 800 °C. The heating rate was maintained at a constant 10 °C min^–1^. A Fourier transform spectrometer (Shimadzu, IRAffinity-1, Kyoto, Japan) operating from 4000 to 400 cm^–1^ was used for FTIR studies, employing the traditional KBr insert procedure. A D8 da Vinci Advance Bruker diffractometer was used for XRD, and radiation with Cu Kα_1_ = 1.54059 Å and Kα_2_ = 1.54443 Å was used. N_2_ adsorption–desorption isotherms and an Autosorb-iQ2 instrument (Quantachrome Instruments, Boynton Beach, FL, USA) were used to calculate the surface area and porosity. The Brunauer–Emmett–Teller equation was used to establish the superficial areas, and pore volumes and sizes were estimated using the Barrett–Joyner–Halenda method. The determination of pH_PZC_ was done using a Mettler Toledo FiveEasy pH/mV bench meter (Columbus, OH, USA) using solutions of NaOH or HCl (both at 1.0 and 0.1 mol L^–1^) to alter the aqueous solutions to the following pH values: 2.5, 5, 7, 9.5, and 11.5. Next, 5 mL of each solution was mixed with 12.5 mg of MMPPy, shaken for 1 min, and allowed to rest for a full day. Using the pH_initial_ versus pH_final_ graph and the pH_PZC_ value, the pH of the solutions was measured once more at the conclusion. Every determination was performed three times (*n* = 3).

### 4.3 MMPPy Synthesis

The synthesis of MMPPy took place in three stages. In the first step, the magnetic nanoparticles (Fe_3_O_4_) were synthesized using 15 mmol of FeCl_3_·6H_2_O and 10 mmol of FeSO_4_·7H_2_O. These reagents were dissolved at 80 °C in 80 mL of ultrapure water while being continuously agitated. After 50 mL of NH_4_OH solution (28%, v/v) was added to the mixture dropwise, it was allowed to sit at 80 °C for 30 min. The Fe_3_O_4_ nanoparticles were gathered, thoroughly cleaned with ultrapure water until the pH was balanced, and then allowed to dry for 24 h at 60 °C in an oven.

The surface of Fe_3_O_4_ was modified with TEOS, obtaining Fe_3_O_4_@SiO_2_, to make it more stable, which has both magnetic properties and surface-active groups. For this, 160 mL of ethanol:ultrapure water (5:1 v/v) and 1.6 g of Fe_3_O_4_ were combined, and the mixture was placed in an ultrasonic bath for 20 min. Subsequently, 10.6 mL of TEOS and 26.7 mL of NH_4_OH (28%, v/v) were quickly added to the prior solution, and the reaction mixture was stirred for 12 h. After that, Fe_3_O_4_@SiO_2_ was extensively cleaned with ultrapure water and dried for 24 h at 60 °C.

MMPPy was obtained using 2.5 g of Fe_3_O_4_@SiO_2_ dissolved in 350 mL of water, exposed to ultrasonication for 5 min and then stirred magnetically for 30 min. Next, the preceding solution was supplemented with 2.0 g of SDS that had been dissolved in 50 mL of ultrapure water. One solution was introduced to the other after it had been stirred and included 16.5 g of FeCl_3_·6H_2_O that had been dissolved in 190 mL of ultrapure water. Lastly, the dropwise addition of 2.5 mL of pyrrole monomer was made. Under continuous stirring, the mixture reacted for 3 h. The resulting black precipitate was dried in an oven at 60 °C after being repeatedly cleaned with ultrapure water.

### 4.4 Human Plasma Samples

The human plasma samples were diluted with 50 mM phosphate buffer (1:1, v/v). After that, 300 μL of sodium hydroxide (1 mol L^–1^) was added to a mixture of 5 mL of human plasma sample and 5 mL of 50 mM phosphate buffer. Following 1 h for protein precipitation in a water bath, the solution was centrifuged for 3 min at 2000 rpm. The supernatant was then extracted, and P4 was added at various concentrations for the purpose of optimizing sample preparation and validating the procedure. After that, NaOH (0.1 mol L^–1^) and HCl (0.1 mol L^–1^) were used to change the pH to various levels in order to assess the sample preparation.

### 4.5 MSPE Procedure

Initially, 10 mg of MMPPy was dispersed in a 1000 μL spiked pool human plasma. After this solution was vortexed for 1 min, the adsorbent was separated with a neodymium magnet (N42 50 × 50 × 5 mm thick, maximum traction equal to 24 kg) and the supernatant discarded. In order to assess applicability, the factors that can influence extraction efficiency were studied, such as the effect of pH, amount of MMPPy, elution solvent, washing solvent, volume of sample and eluent, and reuse. After the extraction phase, the elution solvent was collected and dried under a nitrogen flow. Finally, P4 was resuspended in 50 μL of methanol, and an aliquot of 20 μL was injected to HPLC-UV. The best conditions were chosen through P4 recovery calculated using eq 1. All the analyses were carried out in triplicate (*n* = 3), and the averages were used to plot the graphs for each parameter evaluated.
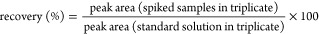
1

### 4.6 Greenness Score

A set of guidelines for assessing the environmental impact of sample preparation and analysis procedures has been provided. We adhered to the standards recommended by Wojnowski and associates in our investigation.^31^ Ten criteria, each with a score ranging from 0 to 1, were evaluated using an open access application and an emulated online version. A final assessment score was also calculated by weighting certain subscores based on their respective values. Greener methods are indicated with higher ratings.^32^

### 4.7 Method Validation

Studies have been performed on selectivity, limit of detection (LOD), limit of quantification (LOQ), linearity, precision, accuracy, robustness, and stability.^31^ By comparing the extracts of pool blank human plasma samples with human plasma samples spiked with P4, we carried out the selectivity assay. By creating the calibration curve (peak area against analyte concentration graphs) with seven P4 concentration levels, namely 5, 500, 1000, 1500, 2000, 2500, and 3000 ng mL^–1^, the linearity was ascertained. The lowest concentration of P4 that could be detected in spiked human plasma samples was used to establish the method’s LOD, and the lowest concentration that could be precisely and accurately determined (below 20%) using six different spiked human plasma samples was used to estimate the LOQ.

The data for precision and accuracy were obtained by the method with the nominal values determined by the low (500 ng mL^–1^), medium (1500 ng mL^–1^), and high (2500 ng mL^–1^) concentrations analyzed in sextuplicate (*n* = 6) in 1 day (intraday) and on different days (interday). The acceptance criterion was defined as the %RSD (precision) and %RE (accuracy) of six determinations (*n* = 6) being less than 15%. Three parameters were varied to determine robustness at 2500 ng mL^–1^: the percentage of the mobile phase (acetonitrile:ultrapure water; 70:30, 72:28, and 68:32, v/v), the flow rate (0.90, 1.00, and 1.10 mL min^–1^), and the chromatography column (Agilent, 250 mm × 4.6 mm, 5.0 μm; Phenomenex, 250 mm × 4.6 mm, 5.0 μm; and Phenomenex, 150 mm × 4.6 mm, 5.0 μm). Lastly, stability tests were accomplished with P4 at two different concentrations (500 and 2500 ng mL^–1^) after 12 h at room temperature (25 ± 3 °C), after 12 h of freeze/thaw cycles, and after freezing for 96 h. The results were compared with the fresh samples using the ANOVA statistical test, adopting a significance level of 95% (*p*-value ≥ 0.05) and precision (%RSD).

### 4.8 Method Application

The Universidade Federal de São João del-Rei Ethics Committee approved the study’s protocol, which was followed in accordance with the Declaration of Helsinki (Project identification code, CAAE number: 20839019.7.0000.5151). Every experiment was carried out in accordance with any applicable rules or regulations. Before taking part in the study, the participants also provided informed consent for inclusion.

The analytical method was developed, validated, and then used to analyze real plasma samples. Four volunteer pregnant women (8, 20, 35, and 37 weeks gestation) provided blood samples for this purpose, which were taken in tubes containing heparin as an anticoagulant. The tubes underwent a 5 min centrifugation at 2000 rpm, and the human plasma samples were examined the same day. The analytical curves were used to determine the analyte concentrations.

## Supporting Information Available

Infrared absorption values; EDS elemental analysis of Fe_3_O_4_, Fe_3_O_4_@SiO_2_, and MMPPy; optimized conditions for MSPE; chromatographic parameters obtained by developed method; precision and accuracy of the analytical method; chromatographic conditions and range investigated during robustness tests; stability test data of P4 in human plasma; pH_PZC_ results; and greenness score data details (PDF)
