# Supplementary material for: Polypyrrole as Adsorbent in Magnetic Solid Phase Extraction for Progesterone Determination from Human Plasma
Source: ACS Omega. 2024 Sep 11;9(38):39904–13. doi: 10.1021/acsomega.4c05456 (PMC11425601; doi:10.1021/acsomega.4c05456)
Supplement: Supplementary file 1 — ao4c05456_si_001.pdf [file ao4c05456_si_001.pdf]

Supplementary material

Polypyrrole as adsorbent in magnetic solid phase extraction for progesterone determination from human plasma

Iara Amorim Carvalho, Camilla Fonseca Silva, Raíra da Cunha and Keyller Bastos Borges*

Departamento de Ciências Naturais, Universidade Federal de São João del-Rei, Campus Dom Bosco, Praça Dom Helvécio 74, Fábricas, 36301-160, São João del-Rei, Minas Gerais, Brazil

*Corresponding author:

Prof. Keyller Bastos Borges, PhD, Departamento de Ciências Naturais, Universidade Federal de São João del-Rei, Campus Dom Bosco, Praça Dom Helvécio 74, Fábricas, 36301-160, São João del-Rei, Minas Gerais, Brazil

*e-mail: keyller@ufsj.edu.br

Phone number.: +55 32 3379 – 5163

Tables

Table S1. Infrared absorption values of Fe_3O_4, Fe_3O_4@SiO_2 and MMPPy.

**Wavenumber / cm^{-1}**	**Assignmentt**
586	Fe-O vibration
1100 / 804	Asymmetric stretching of the Si-O-Si bond
948	Stretch vibration of Si-O
960	Si-OH elongation
3100	Stretching and deformation of the C-H bond of aromatic rings
1506 / 1499	C=C elongation of pyrrole ring
1553 / 1465	Asymmetric and symmetric C-C stretching vibrations of the pyrrole ring
1309	C-N Stretching bond of pyrrole ring
1180	C-C elongation.
1045	C-H vibration bond of pyrrole ring
916	C-C deformation vibration outside the plane of the pyrrole ring

Table S2. Semi-quantitative elemental composition of Fe_3O_4, $Fe_3O_4@SiO_2$ and MMPPy by EDS.

Elements	Fe	C	O	Ti	Si	Al	Total
**Fe_3O_4**	34.67	25.05	38.83	-	-	1.45	100.00
**$Fe_3O_4@SiO_2$**	16.37	-	61.70	-	20.51	1.42	100.00
**MMPPy**	3.79	61.59	23.20	1.80	9.62	-	100.00

Table S3. Optimized conditions for MSPE.

Parameters	Optimized conditions
Washing solvent	Ultrapure water
Washing solvent volume	500 μL
Eluent type	acetonitrile
Eluent volume	1000 μL
Amount of adsorbent (MMPPy)	10 mg
Sample volume	1000 μL
Sample pH	No adjustment
Enrichment factor	20

The eluates were dried, resuspended in 50 μL of methanol and 20 μL submitted to chromatographic analysis.

Table S4. Chromatographic parameters obtained by developed method.

Parameters	Progesterone
t_r / *min*	7.93
k	5.63
Area / %RSD	2.92
N	7725
A_F	1.16

t_r = retention time; k = retention factor (where t_m = 1.7 is defined as the first significant disturbance at the baseline); area (%RSD) = relative standard deviation for analyte retention time and expressed as a percentage; *N* = theoretical plates; A_F= asymmetry factor.

Table S5. Precision and accuracy of the analytical method for the determination P4 in human plasma samples.

Nominal concentration / ng mL^{-1}	500	1500	2500
		**Intra-day / n[a] = 6**	
**Concentration analyzed / ng mL^{-1}**	471.64	1289.3	2376.5
**Precision / %RSD [b]**	7.76	1.55	2.92
**Accuracy / %RE [c]**	-5.67	-14.1	-4.39
		**Inter-day / n[a] = 3**	
**Concentration analyzed / ng mL^{-1}**	464.80	1327.1	2388.9
**Precision / %RSD[b]**	1.60	3.38	0.16
**Accuracy / %RE[c]**	-7.03	-3.69	-4.42

[a] n = number of repetitions; [b] %RSD = mean relative standard deviation; [c] %RE = mean relative error

Table S6. Chromatographic conditions and range investigated during robustness tests.

Variables	Variations	Concentration / ng mL^{-1}	%RSD[a]	%RE[b]	*p*-value[c]
**Flow rate / mL min^{-1}**	0.90	2399.65	3.83	-4.14	0.535
	1.00	2465.23	3.31	-1.39	
	1.10	2478.45	3.37	-0.86	
**Mobile phase proportion / %, *v/v***	70:30	2654.73	4.41	6.19	0.341
	72:28	2580.71	6.57	3.22	
	68:32	2486.56	2.89	-0.53	
**Analytical column**	Agilent (250 mm)	2400.57	4.29	-3.97	0.973
	Phenomenex (250 mm)	2678.65	5.72	-4.85	
	Phenomenex (150 mm)	2393.66	4.77	- 4.25	

[a] %RSD = mean relative standard deviation of the six repetitions; [b] %RE = mean relative error of the six replicates; [c] Significance level of the $p \geq 0.05$

Table S7. Stability test of P4 in human plasma.

	p-value [b]	%RSD [c]
**12 h room temperature (n[a] = 6)**		
500	0.562	3.20
2500	0.132	4.62
**Freeze/thaw cycles (n[a] = 6)**		
500	0.051	4.53
2500	0.137	5.29
**96 h freeze (n[a] = 6)**		
500	0.360	6.21
2500	0.003	4.78

[a] n = number of repetitions; [b] Significance level set at $p \geq 0.05$; [c] %RSD = average relative standard deviation of six repetitions.

Figures

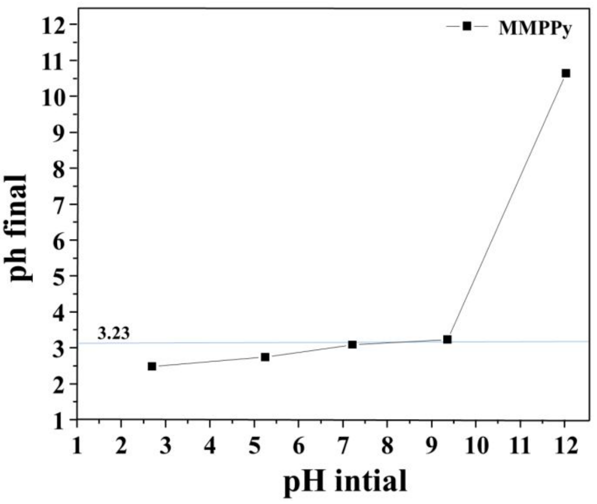

Figure S1. pH_{PZC} of MMPPy.

#	Criterion		Score	Weight
1.	**Sample preparation placement:**	On site	0.33	1
2.	**Hazardous materials:**	1 [g or mL]	0.33	5
3.	**Sustainability, renewability, and reusability of materials:**	Materials are not sustainable or renewable, but are used SEVERAL TIMES	0.50	2
4.	**Waste:**	2.51 [g or mL]	0.48	4
5.	**Size economy of the sample**	Mass or volume of the sample: 1 [g or mL]	0.67	2
6.	**Sample throughput:**	60 [samples/h]	0.96	3
7.	**Integration and automation**	Sample prep. steps: 3 steps, Manual systems	0.19	2
8.	**Energy consumption:**	1 [W]	1.00	4
9.	**Post-sample preparation configuration for analysis:**	GC and HPLC with non-MS detection, atomic absorption spectroscopy, capillary electrophoresis, etc.	0.50	2
10.	**Operator's safety:**	1 hazard	0.75	3

Figure S2. Criterion and data input for each subcategory with the final scores and weight employed for the sample preparation method.
